# Three New Species of *Pyrenula* (Ascomycota, Pyrenulales, Pyrenulaceae) from China

**DOI:** 10.3390/jof12070529

**Published:** 2026-07-18

**Authors:** Xue Li, Lihua Chen, Wenting Liu, André Aptroot, Mingzhu Dou

**Affiliations:** 1 College of Agriculture and Biology, Liaocheng University, Liaocheng 252059, China; 15153845767@163.com; 2 Center of Ecological Forestry Development, Jingning She Nationality Autonomous County, Lishui 323500, China; 15905789649@163.com; 3 School of Mechanical and Automotive Engineering (School of Precision Manufacturing), Liaocheng University, Liaocheng 252059, China; liuwenting@lcu.edu.cn; 4 Laboratório de Botânica, Liquenologia, Instituto de Biociências, Universidade Federal de Mato Grosso do Sul, Campo Grande 79070-900, Brazil; andreaptroot@gmail.com

**Keywords:** lichenized fungi, morphology, phylogeny, taxonomy

## Abstract

The lichenized fungal genus *Pyrenula* Ach. is one of the most common groups of crustose lichens in tropical and subtropical regions, but there has been little research on this genus in China. Based on an integrative taxonomic method including morphological, chemical characters, and three-locus phylogenetic analyses, we found three new *Pyrenula* species from southern China. *Pyrenula falcatispora* sp. nov. is characterized by multiseptate (7–9-septate), falciform ascospores with pointed ends, and the presence of pseudocyphellae; *Pyrenula rectiloculata* sp. nov. is characterized by elongate-fusiform ascospores (5–7(–8)-septate) with pointed ends and rectangular lumina, the absence of pseudocyphellae, and the inspersed hamathecium; *Pyrenula jinghongensis* sp. nov. is unique in having 2-spored asci, large and muriform ascospores with 14–19 transverse septa. Phylogenetic analyses confirmed that these three species form independent lineages within the genus. Detailed descriptions, illustrations, and comparisons with morphologically similar taxa are provided. Notably, taxa with ascospores more than four times as long as wide or 2-spored asci were uncommon within the genus *Pyrenula*. This is the first report of transversely septate *Pyrenula* taxa with ascospores more than four times as long as wide in China. Meanwhile, a key for the *Pyrenula* species reported in China is provided.

## 1. Introduction

*Pyrenula* Ach. is a genus of lichenized fungi, belonging to the family Pyrenulaceae in the order Pyrenulales in the class Eurotiomycetes of the phylum Ascomycota. It was initially established by Acharius with *P. nitida* (Weigel) Ach. designated as the type species [1]. The systematic taxonomy of the genus *Pyrenula* has progressed through several important phases since the last century. Early significant contributions include Harris’s revision of North American species from 1973 to 1989, whose research emphasized the taxonomic value of morphological features such as spore septation and ostiole structure, laying the foundation for modern studies of the genus [2]. Subsequent contributions included global and regional monographs by Aptroot and Upreti [3,4]. A major milestone was reached in 2012 when Aptroot consolidated numerous historical names into 169 accepted species based on type examinations and provided the first comprehensive worldwide identification key for the genus [5]. Weerakoon et al. (2012) first employed molecular phylogenetics to investigate the family Pyrenulaceae, revealing that it comprises two main clades distinguished primarily by the presence or absence of pseudocyphellae, and that the genus *Pyrenula*, as traditionally circumscribed, is not monophyletic [6]. This finding was later confirmed by Gueidan et al. (2016) through additional phylogenetic analyses [7]. Most recently, Schumm and Aptroot (2021) published an updated synthesis of the genus, featuring detailed morphological descriptions, anatomical illustrations (often from type specimens), and a revised global key, marking a significant advance in the taxonomic study of *Pyrenula* [8]. In recent years, several species of *Pyrenula* have also been reported from Asia [9,10,11,12,13,14]. To date, approximately 260 species of *Pyrenula* are known worldwide [5,15,16,17,18,19,20,21,22,23,24], 54 of which have been found in China [21,22,25,26,27,28,29].

*Pyrenula* is characterized by a crustose thallus that is UV− or UV+ yellow, with or without pseudocyphellae, and with or without lichexanthone or anthraquinones. The ascomata are perithecioid, and the hamathecium may occasionally be inspersed with unbranched filaments. Ascospores are transversely septate, or (sub-)muriform; when mature, they are brown with rounded, diamond-shaped, or elongated locules, and may develop orange to red oily droplets in over-mature stages.

Here, we describe three new species, among which two species possess filiform ascospores with more than three transverse septa, and the remaining species is characterized by 2-spored asci and muriform ascospores. These taxonomic conclusions are strongly supported by morphological and molecular phylogenetic analyses.

## 2. Materials and Methods

### 2.1. Morphological and Chemical Analyses

The specimens used in this study were collected from Zhejiang, Yunnan, and Hainan Provinces, and are currently deposited in the Fungarium of the College of Agriculture and Biology, Liaocheng University (LCUF). An Olympus SZX16 dissecting microscope (Olympus Corporation, Tokyo, Japan) was adopted to observe and capture images of morphological characteristics. Anatomical characters were studied and photographically documented using the research microscope Olympus BX53 (Olympus Corporation, Tokyo, Japan) with digital camera Olympus DP74 (Olympus Corporation, Tokyo, Japan). All measurements were performed on mature vertical slices of fruit bodies mounted with water. Measurements are presented as (min–) ($\bar{x}$ − SD) − ($\bar{x}$ + SD) (–max), where ‘min’ and ‘max’ are the observed extreme values, $\bar{x}$ is the arithmetic mean, and SD is the standard deviation [30]. The lichen secondary substances were detected and identified by color test (10% KOH, saturated solution NaClO, and p-phenylene diamine dissolved in 95% ethyl alcohol) and thin-layer chromatography (TLC), using solvent C [31].

### 2.2. DNA Extraction and PCR Sequencing

Genomic DNA was extracted from ascomata using the Hi-DNA-secure Plant Kit (Tiangen, Beijing, China) following the manufacturer’s instructions. Three molecular markers were amplified via polymerase chain reaction (PCR): the internal transcribed spacer region (ITS) using primer pairs ITS1F/ITS4 [32,33]; the nuclear large subunit ribosomal RNA gene (nuLSU) using AL2R/LR6 [34,35]; and the mitochondrial small subunit ribosomal RNA gene (mtSSU) using mtSSU1/mtSSU3R [36]. The total volume of each PCR mixture was set to 50 μL, consisting of 2 μL individual primer stock, 2 μL genomic DNA template, 19 μL ddH_2_O, and 25 μL commercial 2× Taq PCR MasterMix from Tiangen (Beijing, China). PCR was performed under the following thermal cycling conditions: for ITS (ITS1F/ITS4), initial denaturation at 94 °C for 3 min; 35 cycles of 94 °C for 30 s, 52 °C for 30 s, and 72 °C for 1 min 30 s; and a final extension at 72 °C for 10 min. For nuLSU (AL2R/LR6), pre-denaturation at 94 °C for 5 min; 35 cycles of 94 °C for 30 s, 56 °C for 30 s, and 72 °C for 1 min 30 s; and final extension at 72 °C for 10 min. For mtSSU (mtSSU1/mtSSU3R), initial denaturation at 95 °C for 5 min; 35 cycles of 95 °C for 45 s, 50 °C for 1 min, and 72 °C for 1 min 30 s; and final extension at 72 °C for 10 min. Amplification products were visualized on 1% agarose gels stained with ethidium bromide and subsequently sequenced by Tsingke Biotech Co., Ltd. (Beijing, China).

### 2.3. Phylogenetic Analyses

DNA sequences generated in this study were assembled and edited using the program Geneious v.9.0.2 (Biomatters Ltd., Auckland, New Zealand) and then subjected to BLASTn search (https://blast.ncbi.nlm.nih.gov, accessed on 20 January 2025) to check the phylogenetic position. The newly generated sequences were submitted to GenBank (Table 1). First, we downloaded all ITS, nuLSU, and mtSSU sequences of *Pyrenula* from NCBI. To ensure that all taxa were included and to maintain sequence quality as much as possible, when multiple sequences were present for a given molecular marker of the same taxon, we removed sequences shorter than 300 bp. The detailed composition of the final dataset is provided in Table 1. Based on previous studies, *Endocarpon pusillum* and *Cyphellophora europaea* were selected as the outgroup in this study [6,7].

Each marker (ITS, nuLSU, and mtSSU) was independently aligned using MAFFT version 7 [37]. Ambiguously aligned or overly divergent regions were masked with the “maskSegment” function in the R package AlignmentFilter v.1.3.0 [38], setting the stringency-controlling parameter prob to 0.05. Following masking, sites with more than 50% gaps were removed using the “degap” function. We evaluated topological congruence among the three individual gene matrices via the reciprocal bootstrap threshold of 70% [39]. Independent maximum-likelihood phylogenetic reconstructions were conducted for ITS, nuLSU and mtSSU datasets using RAxML v.8.2.12 [40]. Each analysis incorporated 100 bootstrap replicates under the GTRGAMMA substitution scheme and was executed on the CIPRES Science Gateway platform (http://www.phylo.org/portal2/, accessed on 10 March 2026). Topological discrepancies with strong bootstrap support were identified by cross-comparing the three single-gene trees; sequences and operational taxonomic units generating incompatible nodes were subsequently trimmed from the alignments. The three filtered single-locus alignments were then concatenated into a unified supermatrix within PhyloSuite v.1.2.2 [41]. The concatenated data matrix comprised 2363 characters (726 for mtSSU, 707 for ITS and 930 for nuLSU). PartitionFinder 2 was utilized to select optimal substitution models for each gene partition prior to Bayesian inference (BI) [42]. The dataset was partitioned into gene groups, with the GTR+I+G, SYM+I+G and GTR+I+G substitution models applied to mtSSU gene, ITS gene and nuLSU gene, respectively [21]. Bayesian tree construction was implemented in MrBayes 3.2.7 [43]. Two runs of four chains were carried out for 10,000,000 generations and trees were sampled every 1000 generations. The initial 25% of sampled trees were discarded as burn-in samples to exclude pre-convergence topological noise. The maximum likelihood analysis was conducted with the RAxML-HPC 2 on ACCESS v.8.2.12 employing a GTRGAMMA approximation with a rapid bootstrap analysis of 1000 replicates on the CIPRES Scientific gateway portal (http://www.phylo.org/portal2/, accessed on 10 March 2026) for verification [40,44]. ML bootstrap values (BS) ≥ 70% and Bayesian posterior probabilities (PP) ≥ 0.95 were considered as significantly supported. The phylogenetic tree was visualized using Figtree v.1.4.4 and edited in Adobe Illustrator CC2019 software.

## 3. Results

### 3.1. Phylogenetic Results

**Figure 1:**
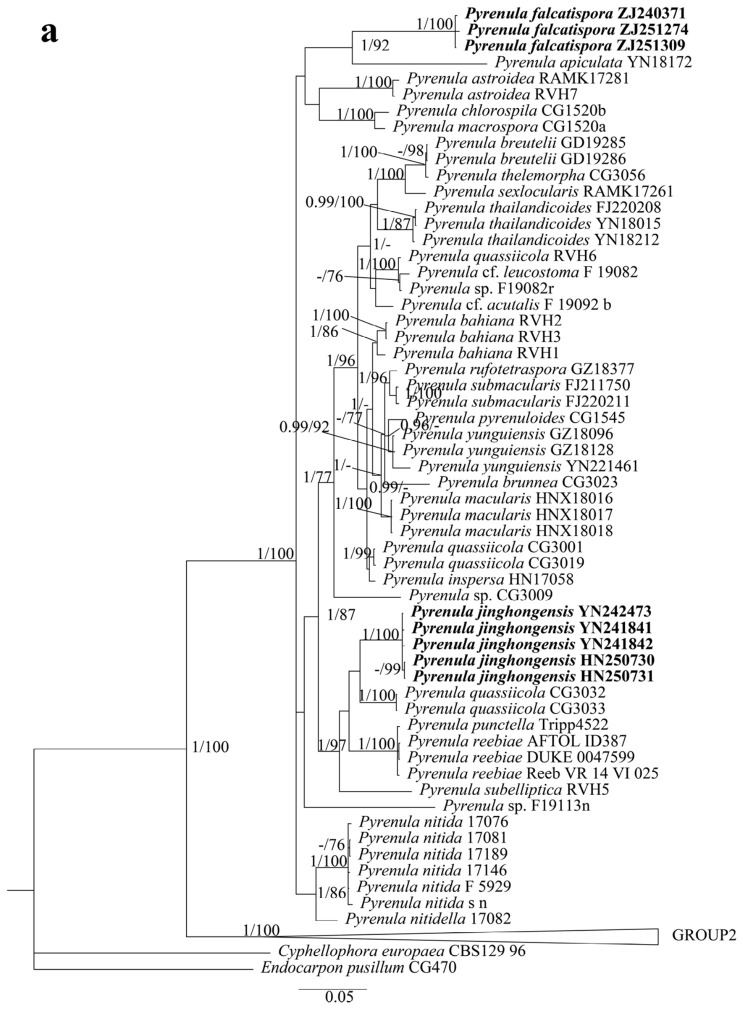

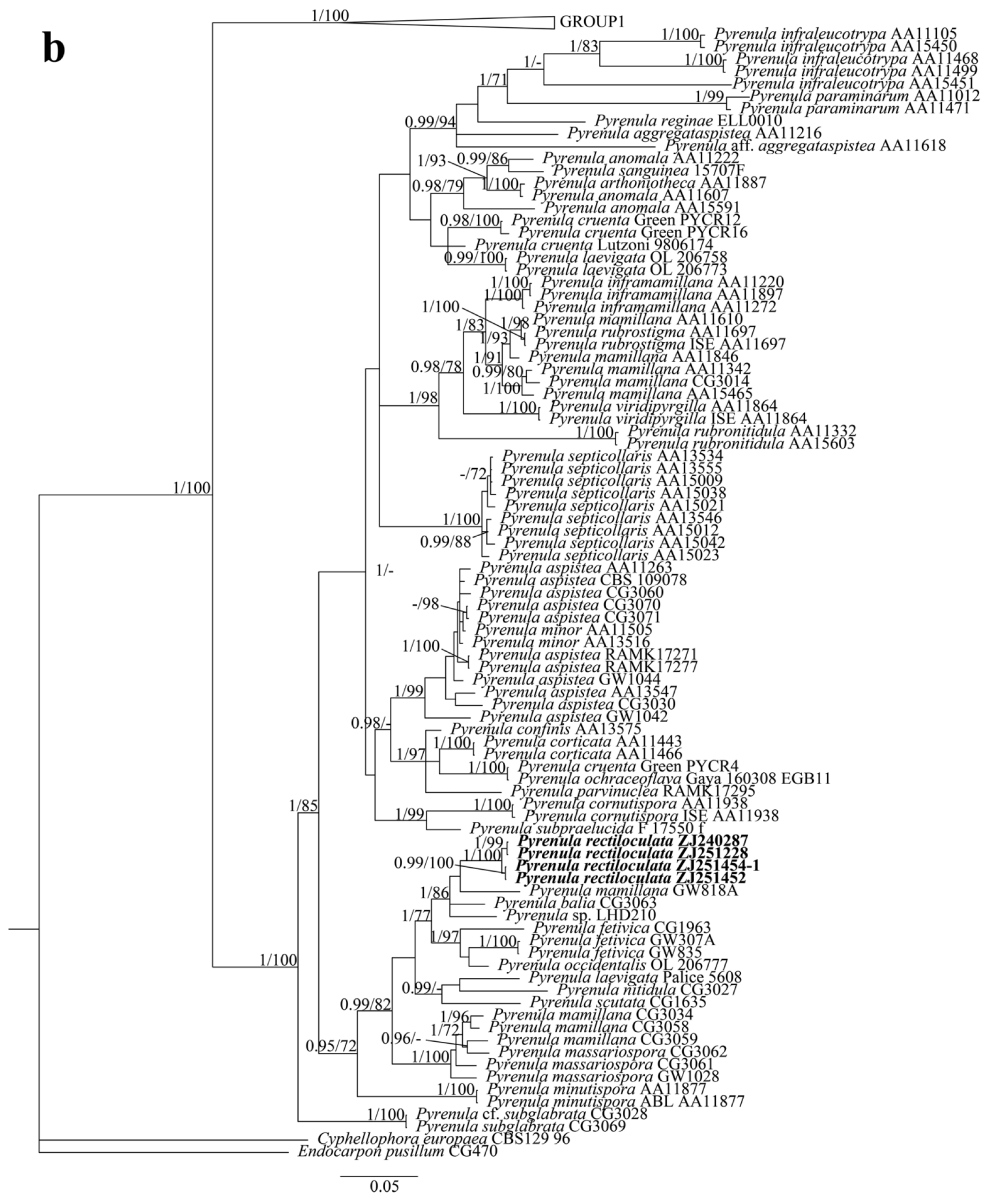
Phylogenetic tree of the genus *Pyrenula* constructed using Bayesian inference based on a three-locus dataset (mtSSU, ITS, and nuLSU). (**a**) Overview of the entire tree and details of Group 1, and (**b**) details of Group 2. Support values (PP/BS) are shown on branches: Bayesian posterior probability ≥ 0.95 (left) and maximum likelihood bootstrap ≥ 70% (right). Hyphen (-) represents support values < 70% BS or <0.95 PP. The new sequences in this study are in bold. Numbers following species names are consistent with the Specimen No. in Table 1.

The dataset comprises 319 DNA sequences (128 ITS sequences, 87 mtSSU sequences, and 104 nuLSU sequences), including 10 ITS sequences, 11 mtSSU sequences, and 10 nuLSU sequences newly generated in this study (Table 1). The phylogenetic trees obtained from the maximum likelihood (ML) and Bayesian inference (BI) exhibit similar topologies, so only the BI tree is provided here (Figure 1). The phylogenetic tree includes two main well-supported monophyletic groups in accordance with previous reports [6,7,21,22].

In the three-locus phylogenetic tree, the specimens of *Pyrenula falcatispora* sp. nov., *P*. *rectiloculata* sp. nov., and *P. jinghongensis* sp. nov. each formed distinct independent clades with high support values (Figure 1). The support values [posterior probability (PP)/bootstrap value (BS)] of the three clades were all 1/100. *P. falcatispora* sp. nov. and *P*. *jinghongensis* sp. nov. were in Group 1, and *P*. *rectiloculata* was in Group 2, coinciding with the presence/absence of pseudocyphellae.

### 3.2. Taxonomy

**Figure 2:**
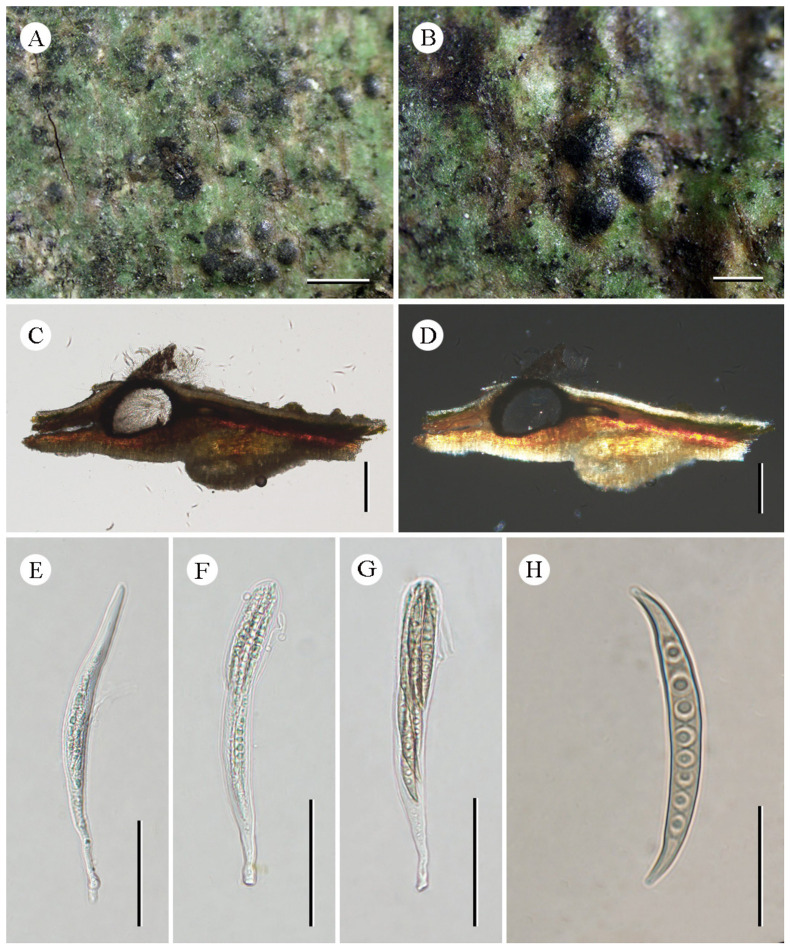
*Pyrenula falcatispora* (LCUF ZJ251274): (**A**) thallus with ascomata, (**B**) ascomata, (**C**) section of an ascoma with surrounding thallus tissue, (**D**) section visualized with polarized light showing crystals, (**E**–**G**) asci, and (**H**) ascospores. Scale bars: 1 mm (**A**), 0.5 mm (**B**), 200 μm (**C**,**D**), 50 μm (**E**–**G**), and 20 μm (**H**).

***Pyrenula falcatispora*** X. Li & M.Z. Dou, sp. nov. (Figure 2).

Fungal Names: FN 573681

**Etymology.** The epithet *falcatispora* refers to the falciform (sickle-shaped) ascospores of this species.

**Diagnosis.** The new species is characterized by falciform ascospores with 7–9 septa. It is similar to *Pyrenula tokyoensis* (Müll. Arg.) H. Harada, but differs in having smaller ascospores and fewer transverse septa.

**Type.** China Zhejiang Province: Lishui City, Jingning County, Dayanghu Nature Reserve, Xiyangkeng Forest District, 27°51′15″ N, 119°45′22″ E, alt. 855 m, on bark, 6 August 2025, M.Z Dou (LCUF ZJ251274—holotype); 7 August 2025, M.Z Dou (LCUF ZJ251309—topotype).

**Description.** Thallus crustose, corticate, dull, brownish-green, with scattered, minute, punctiform pseudocyphellae. Ascomata black, mostly aggregated with fused ostioles, subglobose, convex, partially covered by thalline margins, dehiscing irregularly when overmature, 0.3–0.6 mm. Hamathecium not inspersed, IKI–. Asci 8-spored, ascospores gradually aggregate toward the apex within the ascus as they mature. Ascospores transversely septate with lens-shaped lumina, 7–9-septate, falciform, with acute ends, not tailed, brownish, becoming darker with age, (45–)49–56(–62) × (4.5–)5–6(–6.5) μm ($\bar{x}$ = 52.5 × 5.5 μm, n = 30); lumina subellipsoid to polygonal, end lumina elongated and extending to the spore tips.

**Chemistry.** Thallus K-, C-, KC-, UV-. No substances were detected by TLC.

**Ecology and distribution.** The new species is currently only known from the region of southern China on bark.

**Additional specimens examined.** China Zhejiang Province: Lishui City, Jingning County, Dayanghu Nature Reserve, Xiyangkeng Forest District, 27°51′10″ N, 119°45′42″ E, alt. 860 m, on bark, 3 June 2024, J.C. Li (LCUF ZJ240371).

**Notes.** This new species is similar to *Pyrenula solomonii* A.J. Marshall, de Lange, Blanchon & Aptroot, *P. musaespora* Aptroot & M. Cáceres, *P. subcylindrica* Jagadeesh & Upreti, and *P. tokyoensis* in having multiseptate (more than 3-septate) ascospores. However, *P. solomonii* differs from the new species in having pinkish to pink-gray thallus without pseudocyphellae, larger flattened ascomata (0.5–1.4 mm), fusiform–musiform ascospores, and the presence of pycnidia and conidia [24]. *P. musaespora* is distinguished from the new species by having a grayish-white thallus, fewer transverse septa (3–5-septate), and smaller ascospores (30–37 × 3–4 μm) [45]. *P. subcylindrica* is distinguished from the new species by exospore walls constricted at the septa and the presence of conidia and pycnidia [46]. *P. tokyoensis* is distinguished by its larger ascospores (60–70 × 5–6 μm) and more septa (11–15) [47,48]. Phylogenetically, *P. falcatispora* forms a strongly supported monophyletic clade sister to *P. apiculata* (Figure 1). Despite being its closest relative, *P. apiculata* differs morphologically in having 3-septate ascospores with red or orange oil droplets when over-mature [21].

**Figure 3:**
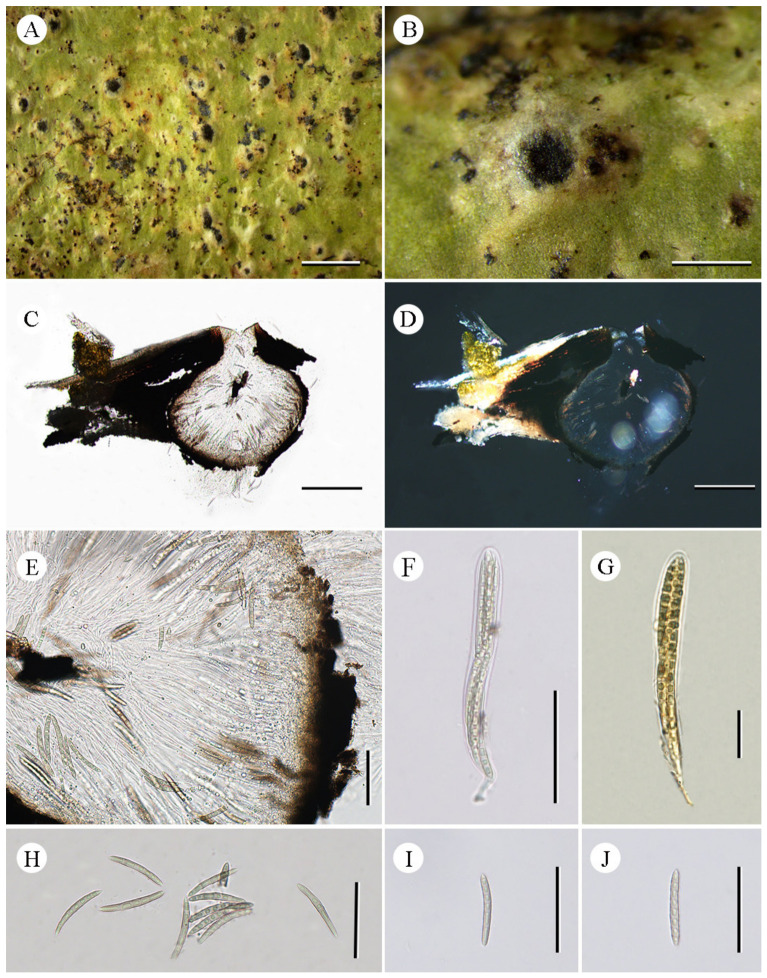
*Pyrenula rectiloculata* (LCUF ZJ240287): (**A**) thallus with ascomata, (**B**) ascomata, (**C**) apothecium section, (**D**) section visualized with polarized light showing cortex of apothecium with crystals, (**E**) inspersed hamathecium, (**F**,**G**) asci, (**F**) in water, (**G**) in IKI, (**H**–**J**) ascospores. Scale bars: 2 mm (**A**), 0.5 mm (**B**), 200 μm (**C**,**D**), 50 μm (**E**,**F**), 20 μm (**G**), and 50 μm (**H**–**J**).

***Pyrenula rectiloculata*** X. Li & M.Z. Dou, sp. nov. (Figure 3).

Fungal Names: FN 573682

**Etymology.** The specific epithet *rectiloculata* is derived from Latin rectus (rectangular) and loculatus, referring to the fact that most lumina of the ascospores are rectangular.

**Diagnosis.** The new species is characterized by elongate-fusiform ascospores with rectangular lumina. It is similar to *Pyrenula moniliformis* (C. Knight) Müll. Arg., but differs in the size of ascospores and the shape of lumina.

**Type.** China Zhejiang Province: Lishui City, Jingning County, Wangdongyang Nature Reserve, Yujikeng Protection Station, 27°41′26″ N, 119°34′53″ E, alt. 960 m, on bark, 2 June 2024, X.Y Luo (LCUF ZJ240287—holotype).

**Description.** Thallus crustose, corticate, pale brownish-green, without pseudocyphellae. Ascomata black, mostly scattered, occasionally aggregated, 0.3–0.8 mm, subglobose, immersed to erumpent, partly covered by the thalline margin. Ostiole apical, black. Hamathecium inspersed with minute granules and colorless oil droplets, IKI+ blue. Ascospores 8 per ascus, 5–7(–8)-septate, elongate-fusiform, slightly acute at both ends, not tailed, brown, (39–)41.5–53(–61) × (3–)4–6(–7.5) μm ($\bar{x}$ = 47 × 5 μm, n = 30); lumina mostly rectangular.

**Chemistry.** Thallus K-, C-, KC-, UV-. No substances were detected by TLC.

**Ecology and distribution.** The new species is currently known only from bark in the subtropical regions of southern China, with the type locality in Jingning County, Zhejiang Province.

**Additional specimens examined.** China Zhejiang Province: Lishui City, Jingning County, Dayanghu Nature Reserve, Xiyangkeng Forest District, 27°51′15″ N, 119°45′22″ E, alt. 855 m, on bark, 6 August 2025, M.Z Dou (LCUF ZJ251228). China Zhejiang Province: Lishui City, Jingning County, Wangdongyang Nature Reserve, Buffer Zone of Yujikeng Protection Station, 27°41′28″ N, 119°34′53″ E, alt. 955 m, on bark, 7 August 2025, X.Y Luo (LCUF ZJ251454-1, ZJ251452).

**Notes.** This new species resembles *Pyrenula largei* A.J. Marshall, de Lange, Blanchon & Aptroot, *P. musaespora* Aptroot & M. Cáceres, *P. subcylindrica* Jagadeesh & Upreti, *P. montagnei* Müll. Arg. and *P. moniliformis* in having ascospores more than 4 times as long as wide. However, *P. largei* possesses more transverse septa (10–18-septate) and larger ascospores (70−100 × 10−15 μm) [24]. *P. musaespora* is distinguished by its grayish-white thallus containing lichexanthone, fewer transverse septa (3–5-septate), and shorter ascospores (30–37 μm) [45]. *P. subcylindrica* differs from the new species in having pseudocyphellae on the thallus and more transverse septa (7–11-septate) [46]. *P. montagnei* differs from the new species in having shorter ascospores (25–38 μm vs. 41.5–53 μm) with transversely subellipsoid lumina [16,49]. *P. moniliformis* is distinguished from this new species by its larger ascospores (55−68 × 10.0−12.5 μm) and moniliform lumina [24,50,51]. Phylogenetically, all specimens of *P. rectiloculata* form a strongly supported monophyletic clade (Figure 1). Although *P. rectiloculata* is sister to *P. mamillana*, this relationship receives low nodal support, and the two species differ significantly in morphological characters. This evidence further confirms the status of *P. rectiloculata* as a new species.

**Figure 4:**
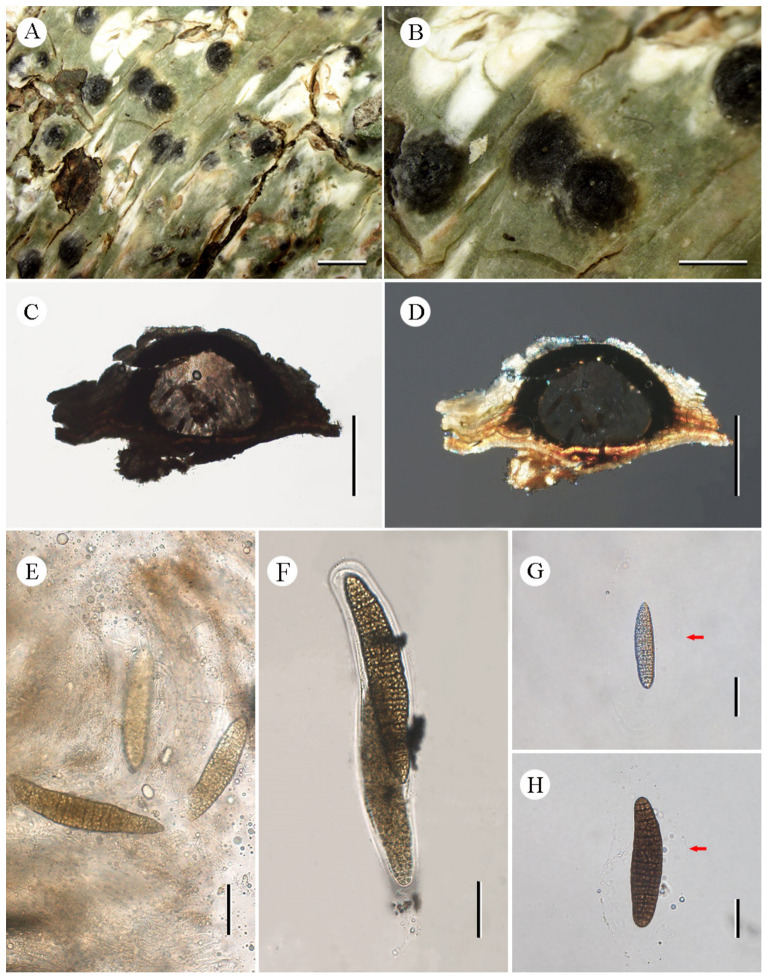
*Pyrenula jinghongensis* (LCUF YN241842): (**A**) thallus with ascomata, (**B**) ascomata and pseudocyphellae, (**C**) apothecium section, (**D**) section visualized with polarized light showing crystals, (**E**) inspersed hamathecium, (**F**) asci, (**G**,**H**) ascospores, with red arrows showing the gelatinous halo. Scale bars: 2 mm (**A**), 1 mm (**B**), 500 μm (**C**,**D**), and 50 μm (**E**–**H**).

***Pyrenula jinghongensis*** X. Li & M.Z. Dou, sp. nov. (Figure 4).

Fungal Names: FN 573683

**Etymology.** The species epithet refers to Jinghong, the locality where the type species was collected.

**Diagnosis.** The new species is characterized by 2-spored asci, muriform ascospores, and inspersed hamathecium. It resembles *Pyrenula globifera* (Eschw.) Aptroot, but differs in having larger ascomata and more septa. It also differs from the morphologically similar *P. platystoma* (Müll. Arg.) Aptroot, *P. neoculata* Aptroot, and *P. duplicans* (Nyl.) Aptroot by its inspersed hamathecium and larger ascospores.

**Type.** China Yunnan Province: Jinghong City, Jiangbei Street, Xiaomo Highway, 21°54′21″ N, 101°10′03″ E, alt. 675 m, on bark, 17 December 2024, X. Li (LCUF YN241842—holotype; LCUF YN241841—isotype).

**Description.** Thallus crustose, corticate, dull, pale green to yellowish-brownish green, with pseudocyphellae. Ascomata black, mostly scattered, occasionally aggregated, with crystals, spherical, prominent, largely covered by the thalline margin; 0.5–1.8 mm. Ostiole apical, brown, surrounded by a ring-like depression. Hamathecium heavily inspersed with hyaline oil droplets, IKI+ red. Asci 2-spored. Ascospores muriform, brown, clavate, with acute to obtuse ends, hyaline when young, becoming brown to dark brown with age, lumina rounded to quadrate, becoming mostly quadrate at maturity, (111–)144–191(–209) × (28–)33–42(–46) μm ($\bar{x}$ = 167 × 37 μm, n = 35), with 14–19 transverse septa and 1–6 longitudinal septa, surrounded by a gelatinous sheath.

**Chemistry.** Thallus K-, C-, KC-, UV-. No substances were detected by TLC.

**Ecology and distribution.** The new species is currently only known from the tropical regions of southern China on bark.

**Additional specimens examined.** China Yunnan Province: Mengla County, Xishuangbanna Tropical Botanical Garden of Chinese Academy of Sciences, Tropical Rainforest, 28°58′17″ N, 101°15′40″ E, alt. 575 m, on bark, 18 December 2024, X. Li (LCUF YN242473). China Hainan Province: Wuzhishan City, The entrance of Wuzhishan National Nature Reserve, 18°53′52″ N, 109°39′46″ E, alt. 710 m, on bark, 13 April 2025, W.Q. Liang (LCUF HN250730, HN250731).

**Notes.** *Pyrenula platystoma* (Müll. Arg.) Aptroot, *P. neoculata* Aptroot, and *P. duplicans* (Nyl.) Aptroot resemble *P*. *jinghongensis* but differ in having a non-inspersed hamathecium and smaller ascospores [25,26,52]. *P. lyoni* (Zahlbr.) Aptroot differs from the new species in having lateral ostioles that are mostly fused [5]. *P. falsaria* (Zahlbr.) R. C. Harris can be distinguished from this species by its lateral, fused ostioles, non-inspersed hamathecium, and 2–6-spored asci [53]. *P. globifera* (Eschw.) Aptroot differs in having smaller ascomata (0.4–0.7 mm) and fewer primary septa (9–15) [53]. *P. xanthoglobulifera* Aptroot, Lücking & M. Cáceres is distinguished by the presence of lichexanthone [54]. Phylogenetically, all specimens of *P. jinghongensis* form a strongly supported monophyletic clade sister to *P. quassiicola* Fée (Figure 1). Despite being its closest relative, *P. quassiicola* differs morphologically in having 8-spored asci and 3-septate ascospores [53].

### 3.3. Key to the Species of Pyrenula Reported in China

Key A (Species with submuriform to muriform ascospores).
Key B (Species with exclusively transversely septate ascospores).
**Key A**
1a. Thallus yellow to orange; anthraquinones pigments K+ pink to purplis…………………………………………………*Pyrenula ochraceoflava* (Nyl.) R.C. Harris
1b. Thallus K– or yellowish, anthraquinones absent…………………………………………2
2a. Ostioles lateral…………………………………………………………………………………3
2b. Ostioles apical…………………………………………………………………………………4
3a. Ascospores < 70 μm long………………………………*Pyrenula astroidea* (Fée) R.C. Harris
3b. Ascospores > 70 μm long………………………………*Pyrenula schiffneri* (Zahlbr.) Aptroot
4a. Ascospores < 25 μm long……………………………………………………………………5
4b. Ascospores > 25 μm long……………………………………………………………………6
5a. Thallus UV + yellow……………………………………*Pyrenula confinis* (Nyl.) R.C. Harris
5b. Thallus UV……………………………………*Pyrenula parvinuclea* (Meyen & Flot.) Aptroot
6a. Old ascospores with orange oil droplets……………………………………………………7
6b. Old ascospores without orange oil droplets………………………………………………11
7a. Ascospores 4/ascus, 70–100(–106) × (17–)21–27(–41) μm, 10–12 × 3–14 locules…………………………………………*Pyrenula rufotetraspora* M.Z. Dou & Z.F. Jia
7b. Ascospores 8/ascus……………………………………………………………………………8
8a. TLC with solvent C showed one spot with fluorescence at Rf five under 365 nm ultraviolet light, ascospores 40–65 × 16–21(–28) um, 7–9 × 2–7 locules…………………………………………*Pyrenula submacularis* M.Z. Dou & Z.F. Jia
8b. TLC with solvent C showed no fluorescent spot at Rf five under 365 nm ultraviolet light………………………………………………………………………………………………9
9a. Most ascospores < 50 μm long……………………………………………………………10
9b. Ascospores 50–70(–80) × 17–22(–26) μm, 8 × 2–4 locules…………………………………………………*Pyrenula yunguiensis* M.Z. Dou & Z.F. Jia
10a. Ascospores (23–)25–37(–41) × (10–)12–15(–18) μm, 8 × 1–4 locules, TLC with solvent C showed no black spot at Rf two under 254 nm ultraviolet light…………………………………………*Pyrenula breutelii* (Müll. Arg.) Aptroot
10b. Ascospores (33–)37–50 × (13–)14–16 μm, 8 × 1–3 locules, TLC with solvent C showed two black spots at Rf two under 254 nm ultraviolet light…………………………………………*Pyrenula macularis* (Zahlbr.) R.C. Harris
11a. Ascospores > 80 μm long, mostly 2/ascus………………………………………………12
11b. Ascospores < 80 μm long, mostly 4–8/ascus……………………………………………15
12a. Thallus without pseudocyphellae, ascospores 80–140(–155) μm long……………………… *Pyrenula platystoma* (Müll. Arg.) Aptroot
12b. Thallus with pseudocyphellae……………………………………………………………13
13a. Hamathecium inspersed, ascospores (111–)144–191(–209) × (28–)33–42(–46) μm…………………………………………*Pyrenula jinghongensis* X. Li & M.Z. Dou
13b. Hamathecium not inspersed………………………………………………………………14
14a. Ascospores 80–110 µm long, 2–4/ascus…………………………………………*Pyrenula neosandwicensis* Aptroot
14b. Ascospores 115–180 μm long, 2/ascus…………………………………………*Pyrenula duplicans* (Nyl.) Aptroot
15a. Locules relatively large and angular, with up to 6 between 2 primary septa…………………………………………*Pyrenula leucostoma* Ach.
15b. Locules mostly round, at least in the central part of the ascospore with more than 6 between 2 primary septa…………………………………………*Pyrenula pyrenuloides* (Mont.) R.C. Harris
**Key B**
1a. Ostioles pointing in various directions, mostly eccentric to lateral; ascomata sometimes with several chambers connected to joint ostioles…………………………………………2
1b. Ostioles apical or, when eccentric, all pointing in the same direction; ascomata with one chamber or each chamber with its own ostiole…………………………………………4
2a. Terminal locules directly against the exospore wall; ascospores 16–25 µm long…………………………………………*Pyrenula circumfiniens* Vain.
2b. Terminal locules separated from the exospore wall by endospore thickening………3
3a. Ascospores 35–45 μm long…………………………………………*Pyrenula adacta* Fée
3b. Ascospores without dark bands, 18–30 µm long *Pyrenula acutispora* Kalb & Hafellner
4a. Ascospores all distinctly 3-septate……………………………………………………………5
4b. Ascospores at least seemingly 4–9-septate…………………………………………………39
5a. Ascospores (45–)50–60 µm long, thallus without papillae but with pseudocyphellae.…………………………………………*Pyrenula immissa* (Stirt.) Zahlbr.
5b. Ascospores mostly < 50 µm long………………………6
6a. Ascomata erumpent, c. 0.4–0.8 mm diam; thallus with red patches or completely red; ascospores 27–35 µm long…………………………………………*Pyrenula cruenta* (Mont.) Vain.
6b. Ascomata and thallus without external pigments…………………………………………7
7a. Ascomata mostly aggregated, with fused walls but with separate ostioles……………8
7b. Ascomata mostly simple, only aggregated by chance when crowded…………………10
8a. Old ascospores with red oil; hamathecium inspersed, ascospores 28.5–50 × 10–20 µm…………………………………………*Pyrenula inspersa* M.Z.Dou & Z.F.Jia
8b. Old ascospores without red oil………………………………………………………………9
9a. Ascospores mostly 21–25 µm long; all locules more or less rounded…………………………………………*Pyrenula leucotrypa* (Nyl.) Upreti
9b. Ascospores mostly 15–20 µm long; ascomata in dense stromata…………………………………………*Pyrenula anomala* (Ach.) Vain.
10a. Thallus ecorticate…………………………………………*Pyrenula laevigata* (Pers.) Arnold
10b. Thallus corticated……………………………………………………………………………11
11a. Old ascospores with orange oil droplets…………………………………………………12
11b. Old ascospores without orange oil droplets………………………………………………15
12a. Ascospores < 35 µm long……………………………………………………………………13
12b. Ascospores > 35 µm long……………………………………………………………………14
13a. Terminal locules directly against the exospore wall…………………………………………*Pyrenula apiculata* M.Z.Dou & Z.F.Jia
13b. Terminal locules separated from the exospore wall by endospore thickening…………………………………………*Pyrenula bahiana* Malme
14a. Hamathecium IKI–; no substances detected by TLC…………………………………………*Pyrenula thailandica* Aptroot
14b. Hamathecium IKI+ red; TLC showed an unidentified substance at Rf four of solvent C…………………………………………*Pyrenula thailandicoides* M.Z.Dou & Z.F.Jia
15a. Terminal locules all directly against the exospore wall…………………………………16
15b. Terminal locules mostly (at least in mature ascospores) separated from the exospore wall by endospore thickening…………………………………………………………………………………………20
16a. Thallus UV+ yellow…………………………………………*Pyrenula pseudobufonia* (Rehm) R.C.Harris
16b. Thallus UV−…………………………………………………………………………………17
17a. Hamathecium inspersed……………………………………………………………………18
17b. Hamathecium not inspersed…………………………………………*Pyrenula nitidula* (Bres.) R.C.Harris
18a. Ascospores all < 16 µm long…………………………………………*Pyrenula cayennensis* Müll. Arg.
18b. Ascospores partly > 16 µm long……………………………………………………………19
19a. Hamathecium inspersed only in the upper part, ascospores at least at one end pointed, biseriate in the ascus…………………………………………*Pyrenula acutalis* R.C. Harris
19b. Hamathecium totally inspersed…………………………………………*Pyrenula fetivica* (Krempelh.) Müll. Arg.
20a. Hamathecium inspersed……………………………………………………………………21
20b. Hamathecium not inspersed………………………………………………………………25
21a. Central ascospore locules elongated…………………………………………*Pyrenula subelliptica* (Tuck.) R.C. Harris
21b. Central ascospore locules transversely lenticular to rounded…………………………22
22a. Ascomata mostly < 0.7 mm diam.; ascospores 18–20 µm long; thallus and ascomata without any anthraquinone……………………………………………*Pyrenula subglabrata* (Nyl.) Müll. Arg.
22b. Ascomata mostly > 0.7 mm diam……………………………………………………………23
23a. Ascospores mostly 10–20 µm long…………………………………………*Pyrenula mamillana* (Ach.) Trevis.
23b. Ascospores mostly > 20 µm long…………………………………………………………24
24a. Ascospores < 30 µm long, ascospores rounded, uniseriate in the ascus…………………………………………*Pyrenula massariospora* (Starb.) R.C. Harris
24b. Ascospores 30–45 µm long, with thin apical walls bulging at the spore tips…………………………………………*Pyrenula supracongruens* Aptroot & Schumm
25a. Thallus UV+ yellow……………………………………………*Pyrenula dermatodes* (Borrer) Schaer.
25b. Thallus UV− or greenish/whitish reflecting………………………………………………26
26a. Ascospores mostly > 25 µm long…………………………………………………………27
26b. Ascospores mostly < 25 µm long……………………………………………………………31
27a. Ascospores 36–45 µm long, without black granules at the tips…………………………………………*Pyrenula subducta* (Nyl.) Müll. Arg.
27b. Ascospores < 40 µm long, without black granules at the tips…………………………28
28a. Ascomata mostly > 0.7 mm diam.…………………………………………………………*Pyrenula complanata* (Mont.) Trevis.
28b. Ascomata mostly < 0.7 mm diam.…………………………………………………………29
29a. Ascospores 32–42 µm long…………………………………………*Pyrenula punctella* (Nyl.) Trevis.
29b. Ascospores mostly 25–37 µm long………………………………………………………30
30a. Central locules much wider than long, ascomata conical, emergent, thallus without pseudocyphellae…………………………………………*Pyrenula mastophora* (Nyl.) Müll. Arg.
30b. Central locules more or less rounded, ascomata somewhat rounded, often partly immersed in the thallus, thallus often with pseudocyphellae…………………………………………………………*Pyrenula quassiicola* (Fée) Fée
31a. Ascospores mostly 21–25 µm long………………………………………………………32
31b. Ascospores mostly < 21 µm long…………………………………………………………34
32a. Ascomata with red, KOH+ purple crystals inside; ascomata > 0.5 mm diam.…………………………………………*Pyrenula nitida* (Weigel) Ach.
32b. Ascomata without KOH+ red crystals……………………………………………………33
33a. Ascospores with angular diamond-shaped locules…………………………………………*Pyrenula micheneri* R.C. Harris
33b. Ascospores with rounded ends…………………………………………*Pyrenula submastophora,* Ajay Singh & Upreti
34a. Ascospores mostly < 15 µm long…………………………………………………………35
34b. Ascospores mostly > 15 µm long…………………………………………………………36
35a. Ascospores 6–8 µm wide…………………………………………*Pyrenula brunnea* Fée
35b. Ascospores 4–6 µm wide…………………………………………*Pyrenula aspistea* (Ach.) Ach.
36a. Ascomata > 0.7 mm diam.…………………………………………………………………37
36b. Ascomata < 0.7 mm diam.…………………………………………………………………38
37a. Locules rounded…………………………………………*Pyrenula scutata* (Stirt.) Zahlbr.
37b. Locules angular…………………………………………*Pyrenula balia* (Krempelh.) R.C. Harris
38a. Ascospores with dark bands between the locules…………………………………………*Pyrenula confoederata* R.C. Harris
38b. Ascospores without dark bands…………………………………………*Pyrenula aggregata* (Fée) Fée
39a. Old ascospores with orange oil droplets, ascospores 5-septate, 22–34 × 8–14 µm…………………………………………*Pyrenula sexlocularis* (Nyl.) Müll. Arg.
39b. Old ascospores without orange oil droplets……………………………………………40
40a. Thallus with pseudocyphellae, ascospore 7–9-septate, falciform, (45–)49–56(–62) × (4.5–)5–6(–6.5) µm…………………………………………*Pyrenula falcatispora* X. Li & M.Z. Dou
40b. Thallus without pseudocyphellae, ascospore 5–7(–8)-septate, lumina mostly rectangular, (39–)41.5–53(–61) × (3–)4–6(–7.5) µm…………………………………………*Pyrenula rectiloculata* X. Li & M.Z. Dou

## 4. Discussion

The genus *Pyrenula* is characterized by widespread phenotypic plasticity and extensive cryptic diversity, making species delimitation based solely on morphological traits often unreliable [6,7]. Accordingly, the integrative taxonomic approach combining morphological anatomy, chemical profiling, and multi-locus phylogenetic data is indispensable for species identification within this genus.

Based on the integrative taxonomic approach, three new species are found and described in detail here. *Pyrenula falcatispora* and *P. rectiloculata* produce transversely septate ascospores that are more than four times as long as wide. *P. jinghongensis* is characterized by 2-spored asci. Taxa with ascospores exhibiting the above traits are uncommon within the genus *Pyrenula*. To date, about 260 species of this genus have been documented worldwide, but only 17 and 9 species possess the above characteristics, respectively [5,15,16,17,18,19,20,21,22,23,24,25,26,51,52,53,54]. The discovery of these new taxa with uncommon traits in China demonstrates that *Pyrenula* in China harbors abundant species diversity and warrants further taxonomic investigations.

The phylogenetic results based on ITS, nuLSU, and mtSSU in this study reveal that the morphologically defined species, such as *P. mamillana*, *P. quassiicola*, and *P. rubrostigma*, are polyphyletic, containing multiple species complexes and cryptic species, which coincides with previous phylogenetic evidence [7,22]. The classification criteria for this group need to be revised. Moreover, gene tree discordance exists on several branches. To further clarify the phylogenetic relationships within this genus, comprehensively employing multiple approaches, such as DNA barcoding, RAD-seq, target-capturing data, multi-gene coalescent phylogenetic analyses, as well as reticulate evolution analysis, should be considered in future studies.

## Figures and Tables

**Table 1:** Information for the sequences used in this study. Newly generated sequences are shown in bold.

Species Name	Specimen No.	Locality	GenBank Accession Number		
			ITS	nuLSU	mtSSU
*P.* cf. *acutalis* R.C. Harris	F_19092_b	Australia	—	DQ329026	DQ329001
*P.* aff. *aggregataspistea* Aptroot & M. Cáceres	AA11618	Brazil Rondônia	—	KT808561	—
*P. aggregataspistea* Aptroot & M. Cáceres	AA11216	Brazil Rondônia	KT820112	KT808557	KT808487
*P. anomala* (Ach.) A. Massal.	AA11222	Brazil Rondônia	KT820168	KT808607	KT808544
*P. anomala* (Ach.) A. Massal.	AA11607	Brazil Rondônia	KT820116	—	KT808490
*P. anomala* (Ach.) A. Massal.	AA15591	Brazil Rondônia	KT820113	—	KT808486
*P. apiculata* M.Z. Dou & Z.F. Jia	YN18172	China Yunnan	OR578592	OR578573	
*P. arthoniotheca* Upreti	AA11887	Brazil Rondônia	KT820120	—	—
*P. aspistea* (Ach.) Ach	AA11263	Brazil Rondônia	KT820121	KT808560	KT808491
*P. aspistea* (Ach.) Ach	AA13547	Brazil Sergipe	KT820123	—	—
*P. aspistea* (Ach.) Ach	CBS_109078	Hong Kong	—	EF411063	—
*P. aspistea* (Ach.) Ach	CG3030	Vietnam Đồng Nai	KT820124	KT808562	KT808494
*P. aspistea* (Ach.) Ach	CG3060	Vietnam Lâm Đồng	KT820125	KT808564	KT808495
*P. aspistea* (Ach.) Ach	CG3070	Vietnam Đồng Nai	KT820126	—	—
*P. aspistea* (Ach.) Ach	CG3071	Vietnam Đồng Nai	KT820127	—	—
*P. aspistea* (Ach.) Ach	GW1042	Sri Lanka Central	JQ927450	JQ927469	—
*P. aspistea* (Ach.) Ach	GW1044	Sri Lanka Central	JQ927451	JQ927470	JQ927462
*P. aspistea* (Ach.) Ach	RAMK17271	Thailand Trat	KT820128	—	KT808492
*P. aspistea* (Ach.) Ach	RAMK17277	Thailand Trat	KT820129	KT808563	KT808493
*P. astroidea* (Fée) R.C. Harris	RAMK17281	Thailand Trat	KT820088	—	—
*P. astroidea* (Fée) R.C. Harris	RVH7	Laos Louangphrabang	KT820089	KT808565	KT808496
*P. bahiana* Malme	RVH1	Laos Louangphrabang	KT820090	—	—
*P. bahiana* Malme	RVH2	Laos Phongsaly	KT820091	KT808614	—
*P. bahiana* Malme	RVH3	Laos Houaphanh	KT820092	KT808605	KT808498
*P. balia* (Kremp.) R.C. Harris	CG3063	Vietnam Lâm Đồng	KT820130	KT808566	KT808499
*P. breutelii* (Müll. Arg.) Aptroot	GD19285	China Guangdong	PP692375	PP692475	—
*P. breutelii* (Müll. Arg.) Aptroot	GD19286	China Guangdong	PP692376	PP692476	PP659692
*P. brunnea* Fée	CG3023	Vietnam Đồng Nai	KT820093	—	—
*P. chlorospila* (Nyl.) Arnol	CG1520b	England	JQ927452	JQ927471	JQ927463
*P. confinis* (Nyl.) R.C. Harris	AA13575	Brazil Sao Paulo	—	KT808567	KT808550
*P. cornutispora* Aptroot & M. Cáceres	AA11938	Brazil Amazonas	KT820131	KT808618	KT808500
*P. cornutispora* Aptroot & M. Cáceres	ISE_AA11938	Brazil Amazonas	NR_158911	NG_060160	—
*P. corticata* (Müll. Arg.) R.C. Harris	AA11443	Brazil Rondônia	KT820132	KT808568	KT808501
*P. corticata* (Müll. Arg.) R.C. Harris	AA11466	Brazil Rondônia	KT820133	KT808569	KT808502
*P. cruenta* (Mont.) Vain	Green_PYCR12	USA	KC592268	—	—
*P. cruenta* (Mont.) Vain	Green_PYCR16	USA	KC592269	—	—
*P. cruenta* (Mont.) Vain	Green_PYCR4	USA	KC592267	—	—
*P. cruenta* (Mont.) Vain	Lutzoni_9806174	Puerto Rico	—	AF279407	—
***P. falcatispora* X. Li & M.Z. Dou**	**ZJ251274**	**China Zhejiang**	**PZ295063**	**PZ295042**	**PZ295052**
***P. falcatispora* X. Li & M.Z. Dou**	**ZJ240371**	**China Zhejiang**	**—**	**PZ295041**	**—**
***P. falcatispora* X. Li & M.Z. Dou**	**ZJ251309**	**China Zhejiang**	**PZ295064**	**PZ295043**	**PZ295053**
*P. fetivica* (Kremp.) Müll. Arg	CG1963	Vietnam Hòa Binh	KT820134	—	KT808503
*P. fetivica* (Kremp.) Müll. Arg	GW307A	Sri Lanka Central	JQ927453	JQ927472	JQ927464
*P. fetivica* (Kremp.) Müll. Arg	GW835	Sri Lanka Central	JQ927454	—	JQ927465
*P. infraleucotrypa* Aptroot & M. Cáceres	AA11105	Brazil Rondônia	KT820114	KT808558	KT808489
*P. infraleucotrypa* Aptroot & M. Cáceres	AA11468	Brazil Rondônia	KT820136	—	—
*P. infraleucotrypa* Aptroot & M. Cáceres	AA11499	Brazil Rondônia	KT820115	—	—
*P. infraleucotrypa* Aptroot & M. Cáceres	AA15450	Brazil Rondônia	KT820142	KT808575	KT808510
*P. infraleucotrypa* Aptroot & M. Cáceres	AA15451	Brazil Rondônia	KT820117	KT808559	KT808488
*P. inframamillana* Aptroot & M. Cáceres	AA11220	Brazil Rondônia	KT820137	KT808572	KT808506
*P. inframamillana* Aptroot & M. Cáceres	AA11272	Brazil Rondônia	KT820138	KT808571	KT808507
*P. inframamillana* Aptroot & M. Cáceres	AA11897	Brazil Amazonas	KT820139	KT808573	KT808508
*P. inspersa* M.Z. Dou & Z.F. Jia	HN17058	China Hainan	OR578591	OR578572	
***P. jinghongensis* X. Li & M.Z. Dou**	**YN241842**	**China Yunnan**	**PZ295065**	**PZ295045**	**PZ295055**
***P. jinghongensis* X. Li & M.Z. Dou**	**YN241841**	**China Yunnan**	**—**	**PZ295044**	**PZ295054**
***P. jinghongensis* X. Li & M.Z. Dou**	**YN242473**	**China Yunnan**	**PZ295066**	**—**	**PZ295056**
***P. jinghongensis* X. Li & M.Z. Dou**	**HN250730**	**China Hainan**	**PZ295067**	**PZ295046**	**PZ295057**
***P. jinghongensis* X. Li & M.Z. Dou**	**HN250731**	**China Hainan**	**PZ295068**	**PZ295047**	**PZ295058**
*P. laevigata* (Pers.) Arnold	OL_206758	Norway Hordaland	MK812685	—	—
*P. laevigata* (Pers.) Arnold	OL_206773	Norway More og Romsdal	MK812185	—	—
*P. laevigata* (Pers.) Arnold	Palice 5608	Slovakia	—	AY607736	AY568029
*P.* cf. *leucostoma* Ach.	F_19082	Australia	—	DQ329024	DQ328999
*P. macrospora* (Degel.) Coppins & P. James	CG1520a	England Devon	JQ927455	JQ927473	JQ927466
*P. macularis* (Zahlbr.) R.C. Harris	HNX18016	China Hunan	PP692368	—	—
*P. macularis* (Zahlbr.) R.C. Harris	HNX18017	China Hunan	PP692369	—	—
*P. macularis* (Zahlbr.) R.C. Harris	HNX18018	China Hunan	PP692370	PP692473	PP659691
*P. mamillana* (Ach.) Trevis.	AA11342	Brazil Rondônia	KT820143	KT808576	KT808515
*P. mamillana* (Ach.) Trevis.	AA11610	Brazil Rondônia	KT820144	KT808615	KT808516
*P. mamillana* (Ach.) Trevis.	AA11846	Brazil Amazonas	KT820145	KT808617	KT808517
*P. mamillana* (Ach.) Trevis.	AA15465	Brazil Rondônia	KT820146	KT808579	KT808519
*P. mamillana* (Ach.) Trevis.	CG3014	Vietnam Đồng Nai	KT820147	KT808580	KT808511
*P. mamillana* (Ach.) Trevis.	CG3034	Vietnam Đồng Nai	KT820149	KT808582	KT808514
*P. mamillana* (Ach.) Trevis.	CG3058	Vietnam Đồng Nai	KT820150	KT808583	KT808518
*P. mamillana* (Ach.) Trevis.	CG3059	Vietnam Đồng Nai	KT820151	KT808584	KT808513
*P. mamillana* (Ach.) Trevis.	GW818A	Sri Lanka Central	JQ927456	JQ927474	JQ927467
*P. massariospora* (Starbäck) R.C. Harris	CG3061	Vietnam Lâm Đồng	KT820153	KT808585	KT808521
*P. massariospora* (Starbäck) R.C. Harris	CG3062	Vietnam Lâm Đồng	KT820154	KT808586	KT808522
*P. massariospora* (Starbäck) R.C. Harris	GW1028	Sri Lanka Central	JQ927457	JQ927475	JQ927468
*P. minor* Fée	AA11505	Brazil Rondônia	KT820155	KT808620	KT808524
*P. minor* Fée	AA13516	Brazil Sergipe	—	KT808587	KT808523
*P. minutispora* Aptroot & M. Cáceres	AA11877	Brazil Rondônia	KT820119	—	—
*P. minutispora* Aptroot & M. Cáceres	ABL_AA11877	Brazil Rondônia	NR_136140	—	—
*P. nitida* (Weigel) Ach.	17076	Poland	MN387114	—	—
*P. nitida* (Weigel) Ach.	17081	Poland	MN387115	—	—
*P. nitida* (Weigel) Ach.	17146	Poland	MN387116	—	—
*P. nitida* (Weigel) Ach.	17189	Poland	MN387117	—	—
*P. nitida* (Weigel) Ach.	F_5929	Czech Republic	JQ927458	DQ329023	DQ328998
*P. nitida* (Weigel) Ach.	s. n.	Germany	—	AY607737	AY568030
*P. nitidella* (Flörke) Müll. Arg.	17082	Poland	MN387139	—	—
*P. nitidula* (Flörke) Müll. Arg.	CG3027	Vietnam Đồng Nai	KT820156	—	KT808525
*P. occidentalis* (R.C. Harris) R.C. Harris	OL_206777	Norway	MK811633	—	—
*P. ochraceoflava* (Nyl.) R.C. Harris	Gaya_160308_EGB11	USA FL	KC592275	—	KC592289
*P. paraminarum* Aptroot & M. Cáceres	AA11012	Brazil Rondônia	KT820135	KT808570	KT808504
*P. paraminarum* Aptroot & M. Cáceres	AA11471	Brazil Rondônia	—	—	KT808526
*P. parvinuclea* (Meyen & Flot.) Aptroot_	RAMK17295	Thailand Trat	—	—	KT808527
*P. punctella* (Nyl.) Trevis.	Tripp4522	—	KT232213	—	KT276274
*P. pyrenuloides* (Mont.) R.C. Harris	CG1545	Vietnam Hà Giang	KT820094	—	—
*P. quassiicola* Fée	CG3001	Vietnam Đắk Lắk	KT820098	KT808588	KT808528
*P. quassiicola* Fée	CG3019	Vietnam Đắk Lắk	KT820101	KT808591	KT808531
*P. quassiicola* Fée	CG3032	Vietnam Đồng Nai	KT820104	KT808592	—
*P. quassiicola* Fée	CG3033	Vietnam Đồng Nai	KT820105	KT808593	—
*P. quassiicola* Fée	RVH6	Laos Phongsaly	KT820107	KT808595	KT808535
***P. rectiloculata* X. Li & M.Z. Dou**	**ZJ240287**	**China Zhejiang**	**PZ295059**	**PZ295038**	**PZ295048**
***P. rectiloculata* X. Li & M.Z. Dou**	**ZJ251228**	**China Zhejiang**	**PZ295062**	**—**	**PZ295051**
***P. rectiloculata* X. Li & M.Z. Dou**	**ZJ251454-1**	**China Zhejiang**	**PZ295060**	**PZ295039**	**PZ295049**
***P. rectiloculata* X. Li & M.Z. Dou**	**ZJ251452**	**China Zhejiang**	**PZ295061**	**PZ295040**	**PZ295050**
*P. reebiae* Aptroot & Gueidan	AFTOL_ID387	USA NC	DQ782845	—	—
*P. reebiae* Aptroot & Gueidan	DUKE_0047599	—	NR_119610	NG_068722	—
*P. reebiae* Aptroot & Gueidan	Reeb VR 14 VI 025	USA NC	—	AY640962	—
*P. reginae* E.L. Lima, Aptroot & M. Cáceres	ELL0010	Brazil Pernambuco	—	KT808596	—
*P. rubronitidula* Aptroot & M. Cáceres	AA11332	Brazil Rondônia	KT820157	KT808597	—
*P. rubronitidula* Aptroot & M. Cáceres	AA15603	Brazil Rondônia	KT820158	—	—
*P. rubrostigma* Aptroot & M. Cáceres	AA11697	Brazil Rondônia	KT820159	KT808616	KT808539
*P. rubrostigma* Aptroot & M. Cáceres	ISE_AA11697	Brazil Rondônia	NR_158913	NG_06015	—
*P. rufotetraspora* M.Z. Dou & Z.F. Jia	GZ18377	China Guizhou	PP692371	PP692474	—
*P. sanguinea* Aptroot, M. Cáceres & Lücking	15707F	Brazil	—	KF697129	KF697128
*P. scutata* (Stirt.) Zahlbr	CG1635	Vietnam Tuyên Quang	KT820160	KT808598	KT808540
*P. septicollaris* (Eschw.) R.C. Harris	AA13534	Brazil Sergipe	KT820166	KT808610	KT808551
*P. septicollaris* (Eschw.) R.C. Harris	AA13546	Brazil Sergipe	KT820161	—	—
*P. septicollaris* (Eschw.) R.C. Harris	AA13555	Brazil Sergipe	KT820167	—	—
*P. septicollaris* (Eschw.) R.C. Harris	AA15009	Brazil Sergipe	—	KT808599	KT808541
*P. septicollaris* (Eschw.) R.C. Harris	AA15012	Brazil Sergipe	KT820162	KT808600	—
*P. septicollaris* (Eschw.) R.C. Harris	AA15021	Brazil Sergipe	KT820163	KT808601	KT808542
*P. septicollaris* (Eschw.) R.C. Harris	AA15023	Brazil Sergipe	KT820164	KT808602	—
*P. septicollaris* (Eschw.) R.C. Harris	AA15038	Brazil Sergipe	—	KT808603	—
*P. septicollaris* (Eschw.) R.C. Harris	AA15042	Brazil Sergipe	KT820165	KT808604	—
*P. sexlocularis* (Eschw.) R.C. Harris	RAMK17261	Thailand Trat	KT820108	KT808606	KT808543
*P.* sp.	F19113n	Australia	—	DQ329027	DQ329002
*P.* sp.	CG3009	Vietnam Đồng Nai	KT820110	KT808611	KT808547
*P.* sp.	F19082r	Australia	JQ927461	DQ329025	DQ329000
*P.* sp.	LHD210	Vietnam	AB935436	—	—
*P. subelliptica* (Tuck.) R.C. Harris	RVH5	Laos Houaphanh	KT820106	KT808594	KT808534
*P. subglabrata* (Nyl.) Müll. Arg.	CG3069	Vietnam Đồng Nai	KT820169	KT808608	KT808545
*P.* cf. *subglabrata* (Nyl.) Müll. Arg	CG3028	Vietnam Đồng Nai	KT820140	KT808574	KT808509
*P. submacularis* M.Z. Dou & Z.F. Jia	FJ211750	China Fujian	PP692372	PP692480	—
*P. submacularis* M.Z. Dou & Z.F. Jia	FJ220211	China Fujian	PP692377	PP692481	—
*P. subpraelucida* Müll. Arg.	F_17550_f	Costa Rica	—	DQ329015	DQ328986
*P. thailandicoides* M.Z. Dou & Z.F. Jia	FJ220208	China Fujian	OR578593	—	—
*P. thailandicoides* M.Z. Dou & Z.F. Jia	YN18212	China Yunnan	OR578589	OR578570	—
*P. thailandicoides* M.Z. Dou & Z.F. Jia	YN18015	China Yunnan	OR578590	OR578571	—
*P. thelemorpha* Tuck.	CG3056	Vietnam Đồng Nai	JQ927460	KT808609	KT808546
*P. viridipyrgilla* Aptroot & M. Cáceres	AA11864	Brazil Rondônia	KT820170	KT808619	KT808548
*P. viridipyrgilla* Aptroot & M. Cáceres	ISE_AA11864	Brazil Rondônia	NR_158914	—	—
*P. yunguiensis* M.Z. Dou & Z.F. Jia	GZ18096	China Guizhou	PP692374	PP692478	—
*P. yunguiensis* M.Z. Dou & Z.F. Jia	GZ18128	China Guizhou	PP692373	PP692479	—
*P. yunguiensis* M.Z. Dou & Z.F. Jia	YN221461	China Yunnan	PP692378	PP692477	—
*Cyphellophora europaea* (de Hoog, Mayser & Haase) Réblová & Unter.	CBS129_96	—	EF551553	FJ358248	FJ225750
*Endocarpon pusillum* Hedw.	CG470	—	JQ927447	EF643754	FJ225677

Notes: Specimen No.: specimen voucher numbers; strain numbers are used if voucher numbers are unavailable in NCBI; isolate numbers are used when neither voucher nor strain numbers are available.

## Data Availability

The original contributions presented in this study are included in the article. Further inquiries can be directed to the corresponding author.
